# Structure-activity relationships of serotonergic 5-MeO-DMT derivatives: insights into psychoactive and thermoregulatory properties

**DOI:** 10.1038/s41380-024-02506-8

**Published:** 2024-03-14

**Authors:** Pol Puigseslloses, Núria Nadal-Gratacós, Gabriel Ketsela, Nicola Weiss, Xavier Berzosa, Roger Estrada-Tejedor, Mohammad Nazmul Islam, Marion Holy, Marco Niello, David Pubill, Jordi Camarasa, Elena Escubedo, Harald H. Sitte, Raúl López-Arnau

**Affiliations:** 1 Department of Pharmacology, Toxicology and Therapeutic Chemistry, Pharmacology Section and Institute of Biomedicine (IBUB), Faculty of Pharmacy, University of Barcelona, 08028 Barcelona, Spain; 2 Pharmaceutical Chemistry Group (GQF), IQS School of Engineering, Universitat Ramon Llull, 08017 Barcelona, Spain; 3 Institute of Pharmacology, Center for Physiology and Pharmacology, Medical University of Vienna, Wäehringerstrasse 13A, 1090 Vienna, Austria; 4 Genetics of Cognition Lab, Istituto Italiano di Tecnologia, Via Morego 30, 16163 Genova, Italy; 5 Hourani Center for Applied Scientific Research, Al-Ahliyya Amman University, Amman, Jordan; 6 Center for Addiction Research and Science, Medical University Vienna, Waehringer Strasse 13A, 1090 Vienna, Austria

**Keywords:** Neuroscience, Psychology, Molecular biology

## Abstract

Recent studies have sparked renewed interest in the therapeutic potential of psychedelics for treating depression and other mental health conditions. Simultaneously, the novel psychoactive substances (NPS) phenomenon, with a huge number of NPS emerging constantly, has changed remarkably the illicit drug market, being their scientific evaluation an urgent need. Thus, this study aims to elucidate the impact of amino-terminal modifications to the 5-MeO-DMT molecule on its interactions with serotonin receptors and transporters, as well as its psychoactive and thermoregulatory properties. Our findings demonstrated, using radioligand binding methodologies, that all examined 5-MeO-tryptamines exhibited selectivity for 5-HT1AR over 5-HT2AR. In fact, computational docking analyses predicted a better interaction in the 5-HT1AR binding pocket compared to 5-HT2AR. Our investigation also proved the interaction of these compounds with SERT, revealing that the molecular size of the amino group significantly influenced their affinity. Subsequent experiments involving serotonin uptake, electrophysiology, and superfusion release assays confirmed 5-MeO-pyr-T as the most potent partial 5-HT releaser tested. All tested tryptamines elicited, to some degree, the head twitch response (HTR) in mice, indicative of a potential hallucinogenic effect and mainly mediated by 5-HT2AR activation. However, 5-HT1AR was also shown to be implicated in the hallucinogenic effect, and its activation attenuated the HTR. In fact, tryptamines that produced a higher hypothermic response, mediated by 5-HT1AR, tended to exhibit a lower hallucinogenic effect, highlighting the opposite role of both 5-HT receptors. Moreover, although some 5-MeO-tryptamines elicited very low HTR, they still act as potent 5-HT2AR agonists. In summary, this research offers a comprehensive understanding of the psychopharmacological profile of various amino-substituted 5-MeO-tryptamines, keeping structural aspects in focus and accumulating valuable data in the frame of NPS. Moreover, the unique characteristics of some 5-MeO-tryptamines render them intriguing molecules as mixed-action drugs and provide insight within the search of non-hallucinogenic but 5-HT2AR ligands as therapeutical agents.

## Introduction

The use of tryptamines has seen an increased growth in recent years, parallel to the growth in “modern shamanism,” a new trend in drug experimentation consisting of the exploration of the inner-self [1]. In this sense, the so-called New Psychoactive Substances (NPS) phenomenon has gained great popularity globally [2]. These substances tend to be analogs of existing controlled drugs or newly synthesized chemicals that mimic the psychoactive effects of substances that are already under control [3–5]. Since their structure slightly differs from their banned analogs, they can be sold without legal implications in certain countries labeled as “research chemicals” [6, 7]. Still, as the illicit market continuously evolves and new tryptamine derivatives appear, there is a lack of comprehensive regulation for these compounds and their legal status often remains unclear.

As for the recreational use of tryptamines, low doses are required to produce psychotropic phenomena, altering sensory perception and mood, and they have been associated with various cases of intoxications and even fatalities [8]. Tryptamines derivatives consist of an indole scaffold, an amino group and an ethyl side chain, resembling the structure of serotonin [9]. Researchers point out that substitution in position 5 of the indole ring is considered to increase potency compared to other substituted and non-substituted tryptamines [10–12], although the clinical effects reported are similar between them [8].

Further, the Psychonaut Project in 2002 revealed that 5-methoxy-substituted tryptamines, such as 5-MeO-DMT or 5-MeO-DALT, were being widely experimented by users [13]. Moreover, other 5-methoxy-substituted tryptamines, such as 5-MeO-DIPT (also called “foxy methoxy”) and 5-MeO-MIPT (“moxy”) have been described in several case studies, reporting fatalities and intoxications [14–16]. Other 5-methoxy analogs such as 5-MeO-MET [17], 5-MeO-NIPT, 5-MeO-EIPT, 5-MeO-MALT [18], and 5-MeO-pyr-T [19] have also appeared on the drug market over the past 10 years.

On the other hand, a renewed interest on the pharmacology of hallucinogens has re-emerged, as new evidence points towards their potential use as therapeutics for treating several mental disorders, such as depression, anxiety, substance abuse and obsessive-compulsive behaviors [20–24]. Despite the growing interest in the therapeutic properties of tryptamines, there are few dose/concentration-response studies in vitro and in vivo that enable a detailed analysis of the mechanisms of action of novel compounds [25], especially 5-methoxy-substituted tryptamines [11].

Serotonin 5-HT2A and 5-HT1A receptors (5-HT2AR and 5-HT1AR) were shown to be the main target of psychedelics, including tryptamine derivatives, being such interaction the main responsible for their hallucinogenic effects [26, 27]. Moreover, other studies have also demonstrated reasonable 5-HT uptake inhibition, or even 5-HT releasing properties for some 5-MeO-substituted and non-substituted tryptamines [28–30]. In fact, mixed action molecules that combine 5-HT uptake inhibition with actions at 5-HT receptors might overcome the limitations of selective serotonin reuptake inhibitors (SSRIs) when treating mood disorders [31–34].

Most of the structure-activity relationship (SAR) studies on tryptamines have focused on substitutions on the indole ring [10, 35–37]. There has, however, been little systematic investigation on the substitution patterns on the terminal amino group. Moreover, previous research has demonstrated that some 5-MeO-tryptamines are able to interact with 5-HT2AR, but with higher affinity and/or potency for 5-HT1AR [29, 35, 38–40], while other authors reported opposite results [28]. Furthermore, literature about the role of 5-HT1AR in the hallucinogenic response induced by psychedelics (i.e., LSD, DOI, tryptamine derivatives) is scarce and oftentimes contradictory [25, 27, 39, 41], as well as their thermoregulatory effects [25, 41–44].

Therefore, the aim of this study was to (i) characterize the pharmacological profile of different amino-substituted 5-MeO-tryptamines at different 5-HT receptors and the serotonin transporter (SERT), using a combination of computational and in vitro assays; (ii) evaluate in vivo their psychedelic effects, thermoregulatory response and locomotor behavior in mice as well as any correlation between both in vivo and in vitro results; (iii) perform a SAR study; (iv) and finally, evaluate the contribution of 5-HT1AR and/or 5-HT2AR to psychedelic effects and thermoregulatory responses in mice.

## Materials and methods

### Subjects

Male Swiss CD-1 mice (Janvier, Le Genest, France) weighing 30–35 g (6–8 weeks old) were used for the behavioral experiments. The animals were housed in polycarbonate cages with wood-derived bedding and temperature-controlled conditions (22 ± 1 °C) under a 12 h light/dark cycle and had free access to food and drinking water. All the studies were carried out following the ARRIVE guidelines [45]. Experimental groups were randomized (block randomization) and researchers were blinded to the group allocation, the outcome assessment and the data analysis. Animal care and experimental protocols were approved by the Animal Ethics Committee of the University of Barcelona under the supervision of the Autonomous Government of Catalonia, in accordance with the guidelines of the European Community Council (2010/63/EU). The sample size was determined using GPower software. The minimal significance was set at 0.05 and statistical power at 0.8.

### Drugs and materials

5-MeO-tryptamines were synthesized as hydrochloride salts (Supplementary Fig. 1), identified and characterized as described in the Supplementary Material. WAY100635 maleate and ketanserin tartrate were obtained from Tocris (Bio-Techne R&D Systems, S.L.U., Madrid, Spain). Solutions for injection were freshly prepared daily in isotonic saline solution (0.9% NaCl, pH 7.4). The radioligands [^3^H]5-HT (33.2 Ci/mmol), [^3^H]ketanserin (22.8 Ci/mmol), [^3^H]8-OH-DPAT (200 Ci/mmol), [^3^H]imipramine (40 Ci/mmol) and the membrane preparations expressing human 5-HT1A and 5-HT2A receptors (h5-HT1AR and h5-HT2AR) were purchased from Perkin Elmer, Inc. (Waltham, MA, USA). *p*-Chloroamphetamine (pCA) and paroxetine were purchased from Sigma Aldrich. All other reagents were of analytical grade and purchased from several commercial sources. See the Supplementary Material for buffers and solutions composition.

### Uptake inhibition, release, and electrophysiology assays with HEK293 cells

#### Uptake inhibition assays

The [^3^H]5-HT uptake inhibition assay was performed as described [46]. See the Supplementary Material for cell culture procedures. The medium was removed from each well and replaced with 200 μL/well of Krebs-HEPES-Buffer (KHB). The cells were pre-incubated for 5 min with different concentrations of drug in KHB at 50 μL/well. Subsequently, the solution was removed and cells were incubated with the drug dilutions and [^3^H]5-HT in KHB for 1 min. The incubation solution was aspirated and the cells were washed with ice-cold KHB and lysed with 1% sodium dodecyl sulfate (SDS). The lysate was collected into vials and scintillation liquid was added. The radioactivity was quantified using a beta-scintillation counter (Perkin Elmer, Waltham, MA, USA). Non-specific uptake was determined in the presence of 30 μM paroxetine. Data were expressed as percentage of control uptake (absence of tryptamine). Assays were carried out per triplicate for at least three independent experiments.

#### Batch release assays

Batch release is a functional radiometric assay to assess transporter-mediated efflux. Cells were preloaded with 0.1 µM [^3^H]5-HT in KHB for 20 min at 37 °C. Subsequently, cells were washed three times with KHB and equilibrated for 10 min in KHB or KHB+monensin (10 µM). Next, cells were incubated with a concentration around and a concentration above their determined IC_50_ values in KHB or in KHB+monensin and the resulting supernatant was transferred to a new well every 2 min. 10 µM pCA and 0.05 µM paroxetine were used as positive and negative controls, respectively. Liquid scintillation cocktail was added both to the wells containing cells (200 µL) and to the transferred supernatant (100 µL). Total radioactivity determined from/in the remaining lysate of the cells plus the transferred supernatant was set as 100%, and the radioactivity determined in each fraction was expressed as a percentage thereof. Assays were performed per duplicate for five independent experiments.

#### Superfusion release assays

Concentration-response release assays were performed using a superfusion assay [47]. Cells were preloaded with 0.1 µM [^3^H]5-HT in KHB for 20 min at 37 °C. Afterwards, cells were manually and gently washed once with KHB, and superfused with KHB at a rate of 0.5 mL/min for 10 min at room temperature (RT), to establish a stable basal release. Three 2 min-fractions of basal release were collected in 10 mL counting vials before exposing cells to various concentrations of 5-MeO-pyr-T (five fractions). Subsequently, the cells were superfused with 1% SDS for three final fractions to determine total radioactivity present at cells at the end of the experiment. 2 mL of scintillation cocktail was added to each vial, radioactivity was measured and expressed as percentage of released radioactivity in relation to the total radioactivity present at the beginning of that fraction. Experiments were performed per triplicate for five independent experiments.

#### Transporter-mediated currents

Whole-cell patch clamping was used to measure the transporter-mediated currents and performed as previously described [31]. In brief, cells were clamped at −60 mV and substrate-induced transporter currents were recorded at RT using an Axopatch 700B amplifier and pClamp 11.2 software (MDS Analytical Technologies, Sunnyvale, CA, USA). Cells were continuously superfused by a DAD-12 superfusion system and an 8-tube perfusion manifold (ALA Scientific Instruments in Farmingdale, NY, USA), which allowed to apply a physiological external solution. The pipette solution mimics the internal ionic composition of a cell. The recorded currents were filtered at 1 kHz and digitized at 10 kHz using a Digidata 1550 (MDS Analytical Technologies) before being analyzed using Clampfit 10.2 software from Molecular Devices located in San Jose, CA, USA. The elicited currents by the test drugs were normalized to the current amplitude elicited by a saturating concentration of 5-HT (10 µM) applied to the same cell. Passive holding currents were subtracted, and the traces were filtered using a 50-Hz digital Gaussian low-pass filter for analysis purposes. Five independent experiments were performed.

### Radioligand binding and calcium mobilization assays

Binding assays were performed as described previously [48, 49]. Briefly, membrane preparations expressing h5-HT1AR, h5-HT2AR or hSERT (see Supplementary Material) were incubated with radiolabeled selective ligands 0.4 nM [^3^H]8-OH-DPAT, 1 nM [^3^H]ketanserin or 3 nM [^3^H]imipramine, respectively. The drugs were diluted in the corresponding binding buffer and tested at increasing concentrations in duplicate. Binding reactions were performed in tubes containing the drug dilutions; 25 μl of the corresponding radioligand; and the membranes (10 μg and 5 μg/500 μL for 5-HT1AR and 5-HT2AR, respectively, and 15 μg/100 μL for hSERT), all diluted in binding buffer. Non-specific binding was determined in the presence of 5-HT (10 μM) for 5-HT receptors; and paroxetine (3 μM) for hSERT and subtracted from total binding. Incubation was performed for 1 h at 27 ^o^C for 5-HT1A/2AR and 22 ^o^C for hSERT, according to the manufacturer’s protocol (Perkin Elmer, Inc., Waltham, MA, USA) and previous studies - saturation and kinetic experiments [48–50], respectively. The binding reactions were stopped by rapid filtration through GF/B glass microfiber filters pre-soaked with 0.5% polyethyleneimine and washing with ice-cold wash buffer. The filters were placed into vials and scintillation cocktail was added. The trapped radioactivity was quantified. Specific binding was defined as the difference between total binding (binding buffer alone) and non-specific binding. Experiments were conducted per duplicate for at least three independent experiments.

5-HT2AR functional assays were performed with CHO/K1 cells expressing human 5-HT2AR using the Invitrogen™ Fluo-4 NW Calcium Assay Kit (Thermo Fisher, Waltham, MA, USA), as described in the manufacturer’s protocol. Briefly, cells were loaded into black 96-well plates with probenecid and the dye provided by the manufacturer. Compounds solutions were added to the wells and fluorescence was quantified through a fluorescence intensity plate reader (VICTOR Nivo Multimode Plate Reader, Perkin Elmer). *E*_max_ was defined as percentage of the response induced by 5-HT (10^−4^ M). All determinations were conducted per triplicate for at least three independent experiments.

### Molecular docking

Structural models for human 5-HT1AR and 5-HT2AR, complexed with 5-HT, were retrieved from the Protein Data Bank, PDBID: 7E2Y [51] and PDBID: 7WC4 [52], respectively. All models were prepared using the QuickPrep protocol implemented in MOE2020 software (Molecular Operating Environment, Montreal, Canada), including the ligand. Molecular docking was conducted using MOE2020 software. The placement method was guided to the interaction site described experimentally by following the template docking protocol, defining the 5-HT indole moiety as scaffold. The GBVI/WSA ΔG score function was applied to quantify the free energy of binding of the docking conformations, setting the total number of conformations to 100. Ligand Efficiency (LE) was calculated as the quotient between S and the number of heavy atoms, to assess the binding affinity independently of the molecular size. The docking protocol was validated by reproducing the interaction mechanism described for 5-HT in the PDB complexes.

### Behavioral studies

#### Head twitch response (dose-response and antagonist experiments)

HTR studies were adapted from [26]. Mice (*N* = 8–10 per group) were injected intraperitoneally (i.p.) with saline or the appropriate tryptamine dose (0.3, 1, 3, 10, or 30 mg/kg) and placed into the observation arena (25 × 25 × 40 cm). A camera mounted above the observation cage recorded the mice for the next 10 min after the injection. Another batch of animals (N = 8–10) received subcutaneous injections of either saline or the respective antagonist (WAY100635 1 mg/kg for 5-HT1AR or ketanserin 1 mg/kg for 5-HT2AR). After a 10-min interval, the animals were i.p. injected with the corresponding tryptamine (10 mg/kg) and placed in the observation arena. Animal behavior was recorded for the next 10 min after the last injection, as mentioned before. Video recordings were examined by two blind observers blinded to the treatments, who assessed the number of head twitches, defined as a rapid rotational jerk of the head which is not contiguous with any grooming behavior [39].

#### Core body temperature measurements

Rectal temperature of mice (*N* = 8–10 per group) was measured 60 min after i.p. injection of the corresponding compound (see previous section “Head twitch response”). This time point was chosen according to the maximal effect observed in pilot experiments. A thermocouple probe (YS451, Panlab, Barcelona, Spain) connected to a digital thermometer (TMP812RS, Panlab, Barcelona, Spain) was inserted 2 cm into the rectum and steady temperature readout was obtained after 10 s of insertion.

#### Horizontal locomotor activity

The HLA test was carried out according to a previous study [53]. The same animals (*N* = 8–10 per group) used for the assessment of HTR were recorded for 30 min in an observation arena, under low-light conditions with white noise. Video recordings were processed using a tracking software (Smart 3.0 Panlab, Barcelona, Spain) to measure the total traveled distance.

### Data analysis

Data were expressed as means±standard deviation (SD). Competition, release and functional assays, and substrate-induced current curves were plotted and fitted by non-linear regression to obtain the IC_50_, EC_50_, and *E*_max_ values. For behavioral studies, data were fitted by non-linear regression and pED_50_ and *E*_max_ values (with the corresponding 95% CI) were estimated by means of a software (GraphPad Prism 10) that further extrapolates the data. The Cheng-Prusoff equation was used to calculate Ki (affinity): Ki = EC_50_/(1 + [radioligand-concentration/Kd]) [54]. HTR curves were fitted using a Gaussian equation as described [55]. Data from batch release assays were statistically analyzed with a mixed-effects model, employing Šidák’s correction for multiple comparisons. Total release at each concentration and the behavioral experiments with the corresponding antagonists were statistically analyzed using one-way ANOVA, followed by Tukey’s post-hoc test. Molecular volume (van der Waals) was calculated using a grid approximation (spacing 0.75 Å). logP was calculated using Molinspiration Cheminformatics software. For in vivo studies, one-way ANOVA with Dunnet’s post-hoc test was used to compare all conditions to saline in dose-response experiments when data were normally distributed. Otherwise, a non-parametric test (Kruskall-Wallis with Dunn’s post-hoc test) was used. Alpha was set at 0.05. Outliers were excluded following ROUT’s method (Q = 0.1%). All statistical analyses and Pearson correlations were conducted using GraphPad Prism 10 software (GraphPad software, San Diego, CA, USA).

## Results

### 5-MeO-DMT derivatives show nanomolar affinity for 5-HT1A receptors and are full 5-HT2AR agonists

**Fig. 1:**
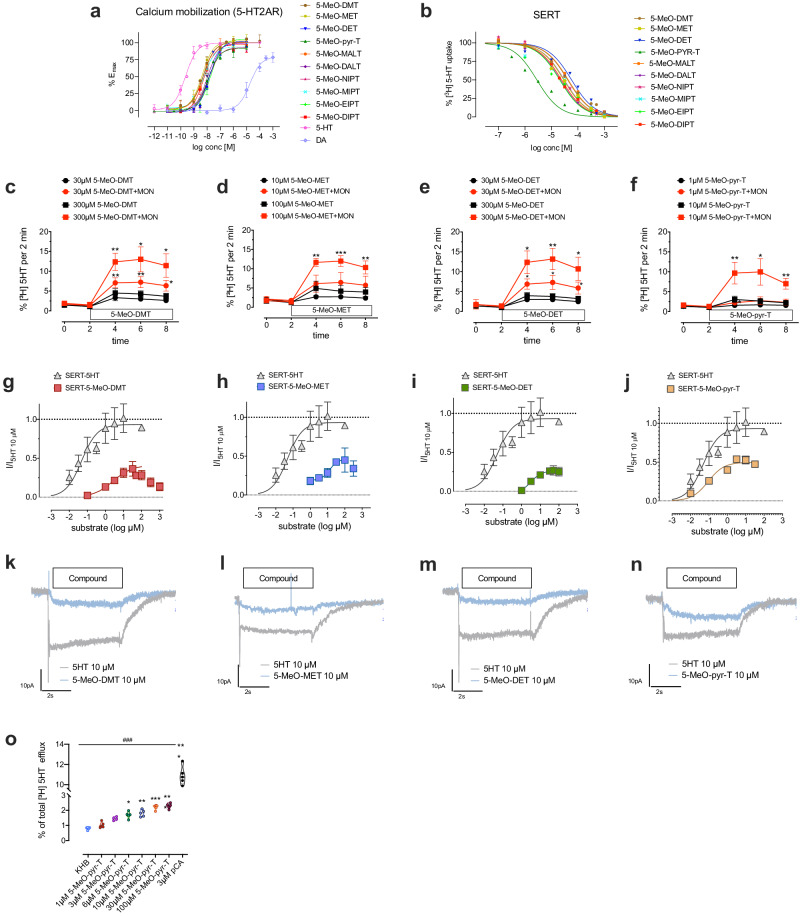
In vitro assays. **a** 5-HT2AR-mediated calcium mobilization assay of the tested 5-MeO-tryptamines and reference compounds 5-HT (full agonist) and dopamine (DA; partial agonist). Data are expressed as means ± SD for *N* ≥ 3 experiments. **b** 5-HT uptake inhibition at SERT. Data are expressed as percentage of control uptake (absence of tryptamine), as means ± SD for *N* ≥ 3 experiments. **c**–**f** Effects of 5-MeO-DMT, 5-MeO-MET, 5-MeO-DET and 5-MeO-pyr-T on transport-mediated batch release of preloaded [^3^H]5-HT from HEK293 cells stably expressing SERT. *p < 0.05, **p < 0.01, ***p < 0.001 vs release in absence of monensin (mixed-effects model, employing Šidák’s correction; *N* = 5). **g**–**j** Whole-cell patch clamp experiments used to identify tryptamine-induced SERT-mediated inwardly directed currents in HEK293 cells (*N* = 5). **k**–**n** Representative single-cell traces showing currents elicited by 10 µM of 5-MeO-DMT, 5-MeO-MET, 5-MeO-DET, and 5-MeO-pyr-T. Data are presented as means ± SD for *N* = 5 independent experiments. **o** Concentration-response relationship of 5-MeO-pyr-T measured in superfusion release assays at different concentrations, as percentage of total efflux (*N* = 5). KHB and pCA were used as control substances. *p < 0.05, **p < 0.01, ***p < 0.001 versus KHB, ^###^*p* < 0.001 vs pCA (Tukey’s test).

The binding affinities for 5-HT1AR, 5-HT2AR, SERT, and EC_50_ and *E*_max_ values for 5-HT2AR-mediated calcium-flux are summarized in Table 1. All the tested compounds displaced [^3^H]8-OH-DPAT and [^3^H]ketanserin binding at nanomolar concentrations (see also Supplementary Fig. 2). Isopropyl-amino compounds (5-MeO-NIPT, 5-MeO-MIPT, 5-MeO-EIPT, 5-MeO-DIPT) showed lower affinity at 5-HT1AR compared to the other compounds tested. In contrast, 5-MeO-pyr-T showed the highest affinity and selectivity for 5-HT1AR. Regarding the affinity for 5-HT2AR, allyl-amino derivatives (5-MeO-MALT and 5-MeO-DALT) showed higher binding affinities. All the tested compounds showed full agonism for 5-HT2AR in calcium mobilization assays, (Fig. 1a). All tryptamines tested displayed low micromolar affinity at SERT, and the obtained Ki values inversely correlated with the volume of the compounds (*R*^2^ = 0.6112, *P* < 0.01; Supplementary Fig. 3).

**Table 1:** Summary of the results obtained for the tested 5-MeO-tryptamines, with their structure and physicochemical properties.

Compounds	5-MeO-DMT	5-MeO-MET	5-MeO-DET	5-MeO-pyr-T	5-MeO-MALT	5-MeO-DALT	5-MeO-NIPT	5-MeO-MIPT	5-MeO-EIPT	5-MeO-DIPT
Chemical structure										
Molecular volume (Å^3^)	237.63	252.00	273.25	262.37	266.38	300.13	255.25	274.50	290.00	308.38
Octanol-water p. coefficient (logP)	2.33	2.71	3.08	2.73	2.97	3.62	2.76	3.00	3.38	3.68
**5-HT1AR**										
**Ki** ± SD (nM)	**2.57** ± 0.09	**3.11** ± 0.51	**4.93** ± 0.62	**0.577** ± 0.195	**5.96** ± 0.47	**3.26** ± 0.39	**15.7** ± 2.3	**24.8** ± 7.6	**18.4** ± 8.4	**15.8** ± 1.3
**5-HT2AR**										
**Ki** ± SD (nM)	**105** ± 22	**94.1** ± 16.7	**128** ± 4	**373** ± 59	**52.9** ± 9	**71.7** ± 14.8	**123** ± 38	**147** ± 32	**151** ± 26	**399** ± 49
**SERT**										
**Ki** ± SD (nM)	**14,510** ± 2925	**7710** ± 3378	**10,410** ± 970	**3006** ± 354	**4015** ± 417	**1189** ± 96	**8590** ± 1706	**2869** ± 928	**1776** ± 83	**1618** ± 475
**5-HT uptake inhibition**										
**IC**_**50**_ ± SD (nM)	**50,068** ± 31,457	**30,102** ± 12,585	**60,012** ± 28,013	**2765** ± 1431	**44,053** ± 11,421	**22,313** ± 4688	**32,110** ± 11,497	**29,768** ± 3918	**22,172** ± 5870	**24,215** ± 1977
**5-HT2AR calcium mobilization**										
**EC**_**50**_ ± SD (nM); **E**_**max**_ ± SD (%5-HT)	**5.28** ± 1.87 **100** ± 5	**4.46** ± 0.06 **102** ± 5	**17.1** ± 5.1 **102** ± 7	**13.5** ± 2.2 **92** ± 4	**4.95** ± 0.47 **99** ± 9	**11.3** ± 3.3 **99** ± 2	**13.2** ± 3.6 **91** ± 1	**5.88** ± 2.39 **96** ± 1	**23.6** ± 1.6 **103** ± 4	**6.21** ± 1.25 **99** ± 6
**HTR**										
**pED**_**50**_ (ED_50_ mg/kg) (95% CI pED_50_) **E**_**max**_ (HTR events) (95% CI)	**0.685** (4.84) (0.499–0.871) **38.1** (31.7–44.5)	**0.435** (2.72) (0.188–0.682) **28.4** (23.5–33.2)	**0.365** (2.32) (0.018–0.711) **9.73** (7.61–11.85)	**0.863** (7.29) (0.626–1.080) **10.0** (6.9–13.1)	**−0.099** (0.796) ((−0.283)–0.085) **27.6** (23.2–32.0)	**0.619** (4.16) (0.419–0.818) **10.4** (8.5–12.4)	**0.686** (4.85) (0.154–1.217) **7.58** (5.46–9.71)	**−0.102** (0.791) ((−0.344)–0.141) **14.19** (11.7–16.68)	**1.03** (10.6) (0.87–1.18) **9.27** (7.10–11.44)	**−0.322** (0.477) −(0.626–0.017) **10.5** (8.5–12.5)
**Change in body temperature**										
**pED**_**50**_ (ED_50_ mg/kg) (95% CI pED_50_) **E**_**max**_ (ΔT ºC) (95% CI)	**0.677** (4.75) (0.307–1.046) **−1.63** −(2.15–1.11)	**0.678** (4.76) (0.268–1.088) **−1.72** −(2.26–1.17)	**0.856** (7.18) (0.697–1.016) **−4.18** −(4.77–3.60)	**0.461** (2.89) (0.257–0.664) **−4.31** −(4.88–3.74)	**1.01** (10.2) (0.80–1.22) **−2.75** −(3.45–2.06)	**1.13** (13.6) (1.04–1.23) **−2.63** −(3.02–2.23)	**0.867** (7.35) (0.720–1.013) **−4.24** −(4.86–3.61)	**1.17** (14.8) (0.951–1.388) **−3.62** −(4.57–2.67)	**1.18** (15.0) (0.991–1.358) **−4.32** −(5.33–3.30)	**1.17** (14.8) (0.93–1.40) **−3.98** −(5.2–2.75)
**HLA** (hypolocomotion) **pED**_**50**_ (ED_50_ mg/kg) (95% CI)	**−0.011 (0.976)** ((−0.327)–0.305)	**0.229 (1.70)** (0.094–0.364)	**0.679 (4.78)** (0.216–1.142)	**0.205 (1.60)** (0.011–0.399)	**0.865 (7.32)** (0.529–1.201)	**0.794 (6.22)** (0.533–1.055)	**0.292 (1.96)** (0.161–0.422)	**0.817 (6.57)** (0.568–1.067)	**0.683 (4.82)** (0.466–0.900)	**0.655 (4.52)** (0.314–0.997)
**E**_**max**_ (% reduction) (95% CI)	**41.72** (32.18–51.26)	**66.79** (58.20–75.37)	**63.56** (37.58–89.53)	**71.25** (60.71–81.79)	**85.40** (55.18–115.6)	**73.11** (57.45–88.78)	**77.64** (69.78–85.50)	**82.78** (62.16–103.4)	**68.02** (54.57–81.46)	**55.32** (38.20–72.44)

Titles and means in bold.

In vitro results for 5-HT1AR, 5-HT2AR, SERT binding affinities, 5-HT2AR-mediated calcium mobilization (*E*_max_ and EC_50_) and 5-HT uptake inhibition (IC_50_). In vivo HTR studies, change in body temperature and HLA. In vitro results are presented as means ± SD for *N* ≥ 3. In vivo results (*N* = 8–10) are presented as *E*_max_ and pED_50_ with 95% CI. ED50 values are also shown (between parentheses) for clarity purposes.

### 5-MeO-DMT, 5-MeO-MET, 5-MeO-DET and 5-MeO-pyr-T act as partial releasers at SERT

Our results demonstrated that all the compounds tested are able to inhibit [^3^H]5-HT uptake in the micromolar range, with 5-MeO-pyr-T displaying the highest potency (Table 1 and Fig. 1b).

Compounds were tested in a release assay using the ionophore monensin (Fig. 1c–f and Supplementary Fig. 4). Monensin causes a rise in intracellular Na^+^ and an alkalization of the interior of the cell, thereby augmenting transporter-mediated efflux [56]. 5-MeO-DMT, 5-MeO-MET, 5-MeO-DET and 5-MeO-pyr-T all showed to be capable of evoking 5-HT release, despite incapable of reaching the same level of efflux triggered by pCA 3 µM (Fig. 1c–f). Followed by the latter, 5-MeO-MIPT and 5-MeO-EIPT evoked a lower release at SERT (<10% total cpm) (Supplementary Fig. 4d, e).

By conducting whole-cell patch clamp experiment on HEK293 cells expressing hSERT, we evaluated the capacity of the four compounds evoking the highest 5-HT release, 5-MeO-DMT, 5-MeO-DET, 5-MeO-MET and 5-MeO-pyr-T, to be transported by hSERT (Fig. 1g–j). All four compounds elicited inwardly-directed currents suggesting their translocation into cytosol. We compared their induced currents to a saturating concentration of 5-HT (10 µM) (Fig. 1k–n). Consistent with previous studies, 5-HT showed an EC_50_ in the low µM range (EC_50_ = 0.05 µM) [57, 58]. 5-MeO-DET produced a blunted current (EC_50_ = 3.5 µM; *E*_max_ = 0.26). 5-MeO-DMT, 5-MeO-MET, and 5-MeO-pyr-T all produced *E*_max_ in the 40–50% range (5-MeO-DMT: *E*_max_ = 0.41; 5-MeO-MET: *E*_max_ = 0.46; 5-MeO-pyr-T: *E*_max_ = 0.49). While 5-MeO-MET showed a moderate activity (EC_50_ = 13 µM), 5-MeO-DMT and 5-MeO-pyr-T showed activity in the low micromolar range (EC_50_ = 2.6 µM; EC_50_ = 0.09 µM, respectively) and partial efficacy compared to 5-HT.

Since 5-MeO-pyr-T was the most potent compound inhibiting 5-HT uptake and elicited 5-HT release at lower concentrations, the potency of 5-MeO-pyr-T to evoke 5-HT release was studied (Fig. 1o), obtaining the following results: EC_50_ = 5.70 ± 1.59 µM (see Supplementary Table 1 for statistical results).

### Molecular docking reveals a better interaction with 5-HT1AR over 5-HT2AR

Tryptamines classically interact with 5-HT1AR and 5-HT2AR. Here, we found a pronounced heterogeneity in the interaction of different derivatives with 5-HT1AR and 5-HT2AR. Therefore, we relied on in silico experiments to establish structure-activity relationships.

**Fig. 2:**
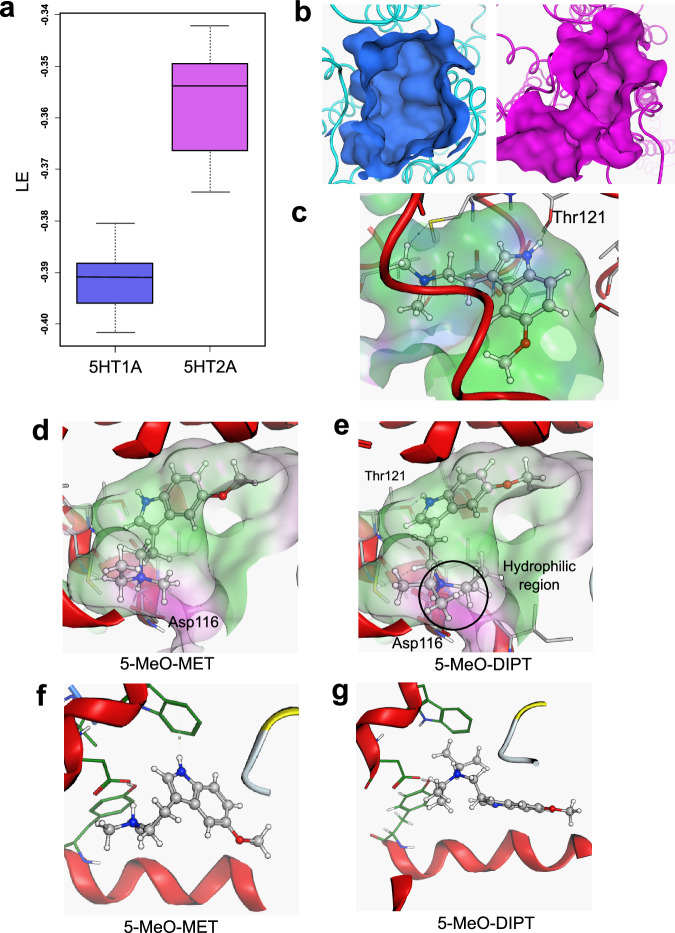
Interaction mechanism of 5-MeO-tryptamines at 5-HT receptors. **a** Ligand efficiency of the compounds when bound to 5-HT1AR or 5-HT2AR. **b** Binding pockets of 5-HT1AR (blue) and 5-HT2AR (purple). **c** Interaction between the indole scaffold and Thr121 of 5-MeO-DMT. **d**, **e** Predicted binding mechanism of 5-MeO-MET and 5-MeO-DIPT at 5-HT1AR. Green zones correspond to hydrophobic regions and purple zones correspond to hydrophilic regions within the pocket. **f**, **g** Spatial orientation of 5-MeO-MET and 5-MeO-DIPT in the 5-HT2AR pocket.

Consistently with our in vitro experiments, molecular docking of tryptamine analogs showed a better interaction of the studied compounds with 5-HT1AR than with 5-HT2AR (Fig. 2a). Although being flexible structures, the analysis of 5-HT1AR and 5-HT2AR binding pockets revealed that entry cavities are significantly different (Fig. 2b).

5-HT1AR shows a bigger cavity, allowing the establishment of a better interaction with the molecules under study in the receptor’s back pocket. For all compounds tested, the indole scaffold reaches the inner part of the receptor and strongly interacts with Thr121 via hydrogen bond (Fig. 2c). The interaction mechanism at 5-HT1AR is stabilized by the formation of one or two hydrogen bonds between the amino group and Asp116 (Fig. 2d). In addition, electrostatic interactions between the positively charged amino-protonated molecules and the negatively charged residue also contribute to the binding affinity, as described for tryptamine binding to 5-HT receptors [59, 60]. Additionally, the arrangement of the alkyl chains from the amino group also plays a role in stabilizing the molecule, e.g., in 5-MeO-MET, the ethyl group is located in a highly hydrophobic region that may contribute to stabilizing the interaction mechanism. Contrarily, although 5-MeO-DIPT shows the interaction with Asp116, the hydrocarbon chains are located in a hydrophilic region (Fig. 2e). This fact may contribute to reducing the binding energy.

Interestingly, the aforementioned interactions between the amino group and the corresponding residue (Asp116 in 5-HT1AR) are greatly reduced when ligands are docked at 5-HT2AR (Fig. 2f–g), preventing a strong interaction with Asp155 [59]. Moreover, the hydrogen bond between the indole moiety and the Thr residue (Thr121 in 5-HT1AR; Thr160 in 5-HT2AR) is non-existent at 5-HT2AR. Attending the crystal structure available in the Protein Data Bank, the electrostatic features of residues in the binding cavity of 5-HT2AR differ from the pattern defined in 5-HT1AR, predictably affecting the recognition process of ligands. In fact, at 5-HT2AR, conformations tend to interact in the solvent-exposed region.

### Potency at 5-HT2AR-mediated calcium-flux correlates to HTR potency

**Fig. 3:**
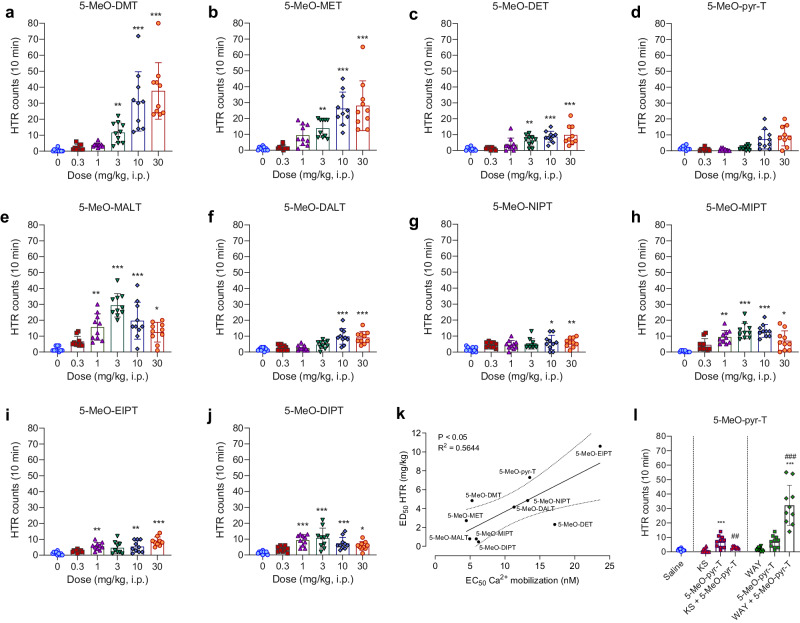
Head Twitch Response. **a**–**j** Number of head-twitch events during a 10-min period for all the tested tryptamines. Data are presented as means ± SD. *p < 0.05, **p < 0.01, ***p < 0.001 vs control group (Kruskall-Wallis with Dunn’s test). *N* = 8–10 mice per group. **k** Correlation between HT2AR-mediated calcium mobilization potency (in vitro) and HTR potency (in vivo), with 95% CI. **l** Representative example of the number of head twitches after 5-MeO-pyr-T injection (i.p., 10 mg/kg) with or without 5-HT1AR or 5-HT2AR antagonist pretreatment, WAY100635 (s.c., 1 mg/kg; WAY) or ketanserin (s.c., 1 mg/kg; KS), respectively. Data are presented as means ± SD. ***p < 0.001 vs control group, ^##^*p* < 0.01, ^###^*p* < 0.001 vs group receiving only 5-MeO-pyr-T (ANOVA with Tuckey’s test). *N* = 8–10 mice per group.

All the 5-MeO-tryptamines tested induced HTR in mice (Fig. 3) with varying potencies (ED_50_) and *E*_max_ values (Table 1). The HTR dose-response profiles followed an inverted U-shape or reached a plateau (Fig. 3a–j and Supplementary Fig. 5). Statistical data are shown in Supplementary Table 2.

Interestingly, we found a correlation between in vitro 5-HT2AR-mediated calcium mobilization potency and HTR potency (*P* < 0.05, *R*^2^ = 0.5644; Fig. 3k) and *E*_max_ (*P* < 0.05, *R*^2^ = 0.4194; Supplementary Fig. 6). In addition, the smaller the size of the tryptamines (e.g., 5-MeO-DMT, 5-MeO-MET) the higher was the HTR observed (*P* < 0.05, *R*^2^ = 0.4082; Supplementary Fig. 7).

### 5-HT1AR modulates both psychedelic and hypothermic effects

To evaluate for target specificity, we also evaluated the HTR response elicited by tryptamine derivatives following a pretreatment with the selective 5-HT2AR antagonist ketanserin. Consistent with a dependence of HTR to 5-HT2AR agonism [39], administering ketanserin prior to tryptamine injection prevented HTR response. However, when administering the selective 5-HT1AR antagonist WAY100635 followed by tryptamine injection, the number of head twitches increased significantly compared to tryptamine derivative alone. Figure 3l shows the results obtained for 5-MeO-pyr-T as a representative example. Results and statistical data for all 5-MeO-tryptamines are presented in Supplementary Fig. 8 and Supplementary Table 3, respectively.

**Fig. 4:**
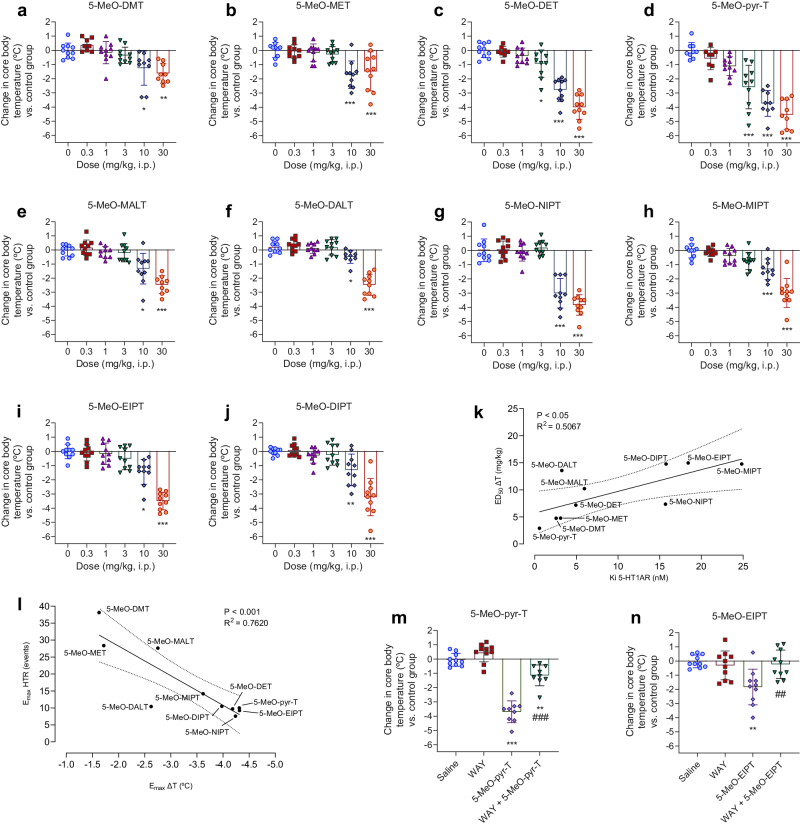
Hypothermic response. **a**–**j** Change in core body temperature 60 min post injection. Data are presented as means ± SD. *p < 0.05, **p < 0.01, ***p < 0.001 vs control saline group (ANOVA with Dunnet’s test or Kruskall-Wallis with Dunn’s test). *N* = 8–10 mice per group. **k** Correlation between affinity for 5-HT1AR (in vitro) and potency in the hypothermic response (in vivo). **l** Correlation between maximal effects in the hypothermic response and HTR. Discontinuous lines represent 95% CI. **m**, **n** Representatives examples (5-MeO-pyr-T and 5-MeO-EIPT, i.p., 10 mg/kg) of the core body temperature measured after 60 min of tryptamine injection with or without WAY100635 pretreatment (s.c., 1 mg/kg; WAY). Data are shown as means ± SD. **p < 0.01, ***p < 0.001 vs control saline group. ^##^*p* < 0.01, ^###^*p* < 0.001 vs group receiving only tryptamine (ANOVA with Tuckey’s test). *N* = 8–10 mice per group.

Regarding the thermoregulatory effects induced by 5-MeO-tryptamines, there is a significant decrease in core body temperature measured 60 min after injection (Table 1 and Fig. 4a–j). Dose-response curves and statistical data are provided in the Supplementary Material (Supplementary Table 2 and Supplementary Fig. 9**)**. A correlation exists between in vivo potency at inducing hypothermia and in vitro binding affinity at 5-HT1AR (*P* < 0.05, *R*^2^ = 0.5067; Fig. 4k). Moreover, we found an inverse correlation between maximal effects of HTR and hypothermic response (*P* < 0.001, *R*^2^ = 0.7620; Fig. 4l).

When administering WAY100635 prior to tryptamine injection, the decrease in body temperature was lower compared to the group receiving the tested compound alone. Figure 4m, n shows the results obtained for 5-MeO-pyr-T and 5-MeO-EIPT as representative examples. Results derived from the core body temperature study for all the 5-MeO-tryptamines are presented in Supplementary Fig. 10. Statistical data are shown in Supplementary Table 3.

### 5-MeO-DMT derivatives induce hypolocomotion

**Fig. 5:**
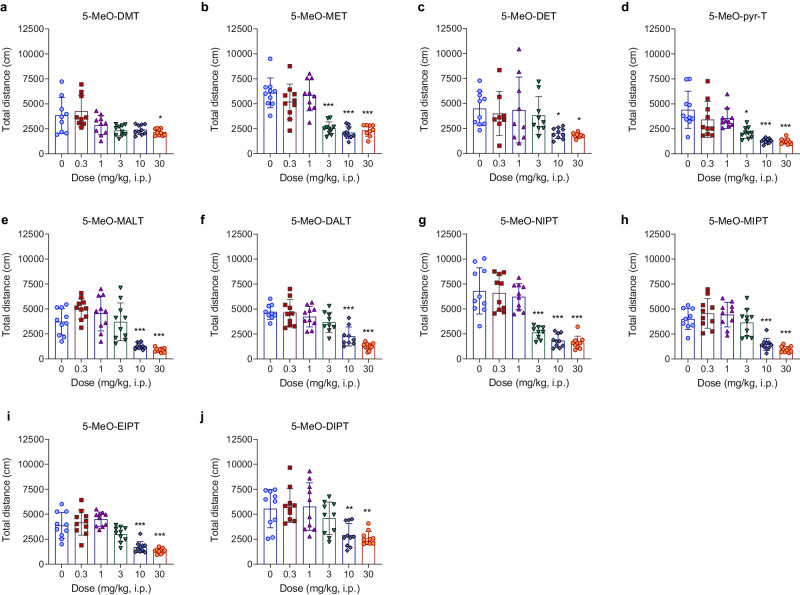
Horizontal locomotor activity. **a**–**j** Total distance traveled in a 30-min period. Data are presented as means ± SD. *p < 0.05, **p < 0.01, ***p < 0.001 vs control saline group (ANOVA with Dunnet’s test or Kruskall-Wallis with Dunn’s test). *N* = 8–10 mice per group.

5-MeO-tryptamines induced a dose-dependent decrease of HLA in mice (Fig. 5). Table 1 summarizes the hypolocomotion potencies for all tryptamines tested. Dose-response curves and statistical data are shown in Supplementary Fig. 11 and Supplementary Table 4, respectively. Hypothermic response and hypolocomotion potencies are correlated (*P* < 0.01, *R*^2^ = 0.5904; Supplementary Fig. 12**)**.

## Discussion

As the NPS market continuously evolves and new cases related to new synthetic tryptamines emerge, it is crucial for health and legal authorities to continue monitoring and address the effects associated with their recreational use [3, 4]. Thus, our study intends to provide useful information to the implicated organisms by characterizing novel 5-MeO-tryptamines through a SAR study based on the N,N-substitutions of the synthetic tryptamines, which may also offer valuable data to predict the pharmacological effects of structurally similar tryptamines that might appear in the future. In addition, a better understanding of the pharmacological profile of this class of tryptamines will offer valuable knowledge for the potential use of these compounds as promising therapeutical agents [21, 52, 61].

One of the aims of the present study was to explore how specific N,N-alkyl and N,N-allyl substitutions on 5-MeO-tryptamines affect the key 5-HT receptors responsible for regulating psychedelic effects, along with their interaction with SERT. Although all tested 5-MeO-tryptamines showed nanomolar affinity for 5-HT1AR, we found that 5-MeO-tryptamines with isopropyl-terminal amino groups showed lower affinities compared to the rest of the molecules, in line with previous studies [62]. The binding mode of all molecules was examined by means of docking calculations, reproducing a strong interaction between the indole scaffold and the Thr121 in the inner part of 5-HT1AR, and also the critical placement of the amino group interacting with Asp116. Hydrophobic interactions may also contribute to stabilization of the molecule in the binding pocket. The loss of these interactions with isopropyl derivatives may explain the lower affinity of these tryptamines.

In contrast to 5-HT1AR, the binding mechanisms predicted for all compounds at 5-HT2AR did not show a key interaction. The change in the pocket’s volume would allow free movement of the ligand, hampering the recognition and the fit of the ligand to the binding cavity. Moreover, the electrostatic features of residues in the binding cavity of 5-HT2AR differs from the pattern defined in 5-HT1AR, predictably affecting the recognition process of ligands. These in silico predictions are in accordance with our in vitro findings, which reveal a higher affinity for 5-HT1AR over 5-HT2AR for 5-MeO-tryptamines, as previously described in the literature for several tryptamine derivatives [63–65]. Although our findings show that the length of the alkyl chains does not have an impact on the affinity for 5-HT2AR, as previously described [36, 62], our binding results suggest the presence of N, N-allyl groups increase affinity for this receptor.

Regarding SERT interaction, steric effects seem to have an impact on the affinity for such transporter, as suggested previously for different molecules [66, 67]. In fact, we found a significant correlation between the size of the molecule and the experimental Ki values: molecules with bulkier substituents on the N position possess higher affinities for SERT. Similarly, a study on quaternary ammonium salts of 4-substituted tryptamines [68] reported increase in affinity for SERT binding and potency for 5-HT uptake inhibition when increasing the bulk of the ammonium unit.

Our results demonstrated that some tryptamines were able to promote 5-HT transported-mediated release, with 5-MeO-DMT, 5-MeO-MET, 5-MeO-DET and 5-MeO-pyr-T showing significant releasing properties among the tested compounds. The slight to none 5-HT releasing activity observed for 5-MeO-DIPT, 5-MeO-MIPT, and 5-MeO-DALT is in accordance with those previously reported [29]. In fact, it is known that large molecules targeting particular monoamine transporters face steric interactions and tend to result in pure blockers, while smaller compounds tend to be better releasers as they can be transported through the membrane more easily [28, 69, 70]. Some authors [28] also suggested that addition of 5-methoxy groups to the chemical scaffold of tryptamines resulted in less active 5-HT releasers, which could explain the weak releasing properties observed for the tested compounds. Since monoamine transporters use the sodium gradient across cell membranes to concentrate their substrates in the cytosol [60], electrophysiology experiments are a useful tool to identify substrates [71]. Therefore, whole-cell patch clamp experiments were performed to study the capacity of 5-MeO-DMT, 5-MeO-MET, 5-MeO-DET and 5-MeO-pyr-T to elicit SERT-mediated inward current. Our results showed that the tested compounds are partial SERT substrates, with 5-MeO-pyr-T being the most potent. Concentration-response release assays were subsequently performed for 5-MeO-pyr-T to assess the 5-HT releasing potency of this compound. The significant difference between the peak of efflux caused by 5-MeO-pyr-T and the positive control *p*CA allows the classification of this tryptamine as a 5-HT partial releaser, a mechanism that is gaining a lot of interest for their potential therapeutic use [31, 72–74]. Although it does not seem that 5-HT releasing is the prime mechanism of the tested compounds, it may still play a supportive role. Moreover, the likely interaction with 5-HT1A autoreceptors (negative inhibitory feedback) may play an opposite role in the increased 5-HT levels at the synaptic cleft, but not in the transporter-mediated efflux itself. Therefore, further studies are needed in order to corroborate if such 5-HT releasing mechanism has implications in the likely in vivo therapeutical effects of novel psychedelics/tryptamines.

All the tested tryptamines induced HTR in mice, as a measure of its potential hallucinogenic effects in humans [75]. This psychedelic response is known to be mainly triggered after activation of 5-HT2AR [26, 27], as ketanserin (5-HT2AR antagonist) pretreatment completely blocks the head twitch behavior. In general, small amino substituents in 5-MeO-tryptamines tend to produce more head twitches, existing a correlation between molecular volume and HTR efficacy. Since some tryptamines showed low activity in the HTR, we tested the in vitro functionality at 5-HT2AR through calcium mobilization assays. Our results showed that all the tested 5-MeO-tryptamines act as full agonists at 5-HT2AR. Yet, potencies vary from one compound to another, correlating to both potency and efficacy in eliciting HTR: potent compounds inducing in vitro calcium mobilization tend to exhibit higher potency and higher efficacy in the HTR. On the other hand, 5-HT2AR agonists that produce little or no hallucinogenic effects are gaining a lot of interest due to their potential as antidepressants [76]. In this sense, 5-MeO-DIPT, 5-MeO-NIPT, 5-MeO-EIPT, and especially, 5-MeO-pyr-T induce very low HTR but still have 5-HT2AR-mediated calcium mobilization potency at nanomolar range. Further studies are needed to elucidate the mechanisms that explain these particularities (i.e., beta-arrestin versus Gq pathways after 5-HT2AR activation [77, 78]). Moreover, recent studies demonstrate that lipophilic tryptamines (e.g., 5-MeO-DMT) exhibit greater abilities to promote neuroplasticity, a key factor in the treatment of mood disorders, due to intracellular 5-HT2AR activation [61]. Therefore, some of the compounds tested in the present study, which are more lipophilic than 5-MeO-DMT and produce low HTR, could be promising candidates for future studies focused on their neuroplasticity properties, with the aim of searching novel psychedelics for treating mood disorders.

Another important site of action for tryptamine derivatives is 5-HT1AR [65, 79, 80]. It has been proposed that 5-HT1AR ligands can modulate 5-HT2AR-mediated effects [42, 81]. For example, some studies describe that pretreatment with the 5-HT1AR agonist 8-OH-DPAT attenuates HTR [27, 82]. Yet, the role of 5-HT1AR in the hallucinogenic effects is still object of debate and controversy. Fantegrossi and coworkers [39, 83] used the 5-HT1AR selective antagonist WAY100635 prior to injection of 5-MeO-DIPT and N,N-DPT, and described a partial attenuation in the HTR for both tryptamine compounds. A more recent study [25] reported no enhancing or inhibition in the HTR when pretreating with WAY100635 followed by psilocyn administration. Contrarily, Glatfelter and coworkers [41] recently reported the 5-HT2AR-mediated HTR is attenuated by 5-HT1AR agonist activity. The discrepancy between the different findings concerning the functional interaction between 5-HT1AR and 5-HT2AR could be attributed to the use of different doses of WAY100635, mouse strain and/or the tryptamine derivative itself. In line with the latter observations, our results indicate that pretreating animals with the 5-HT1AR antagonist followed by administration of 10 mg/kg of tryptamine (the dose in which the HTR effects are significant and close to the *E*_max_) induce a significant increase in the HTR, which points out that 5-HT1AR activation attenuates the HTR behavior. Confirming the role of 5-HT1AR in the psychedelic experience, Pokorny and coworkers [81] found out that 5-HT1AR agonists such as buspirone reduce psilocybin-induced symptoms in humans, including visual hallucinations, derealization and depersonalization, via activation of 5-HT1AR and/or an interaction between 5-HT1A and 5-HT2A receptors, suggesting particular 5-HT1AR agonists could be useful for the treatment of schizophrenia and visual hallucinations in Parkinson’s disease.

Several studies have reported a hyperthermic effect on rodents and humans induced by hallucinogenic drugs, suggesting the 5-HT2AR involvement in the raise of body temperature [43, 84, 85]. Nevertheless, 5-MeO-tryptamines have also a high affinity for 5-HT1AR, a receptor also known to be involved in central body temperature regulation [86–88]. In fact, 5-HT1AR agonists have been reported to cause a dose-dependent hypothermia in rodents [89]. In an attempt to further characterize the pharmacological profile of 5-MeO-tryptamines, we monitored core body temperature after drug administration. Our results showed a dose-dependent hypothermic effect on core body temperature, in agreement with previous studies [42]. The use of WAY100635 (5-HT1AR antagonist) for pretreating mice attenuated the significant hypothermic effects induced by a dose of 10 mg/kg of the corresponding tryptamine, thus confirming the implication of 5-HT1AR on temperature regulation, as reported previously [25, 41, 42]. In fact, we found that high affinity for 5-HT1AR correlates with greater potencies at inducing hypothermic effects in vivo.

As mentioned before, activation of 5-HT1AR can cause a decrease in the 5-HT2AR-mediated effects. In line with this, our in vivo observations showed an inverse correlation between the HTR and hypothermic maximal effects. This finding further corroborates the opposite role of both 5-HT receptors: 5-MeO-tryptamines that produce more 5-HT1AR-related effects (hypothermia) induce less psychedelic-like effects through 5-HT2AR.

All the tested 5-MeO-tryptamines decreased HLA in mice, as previously reported for other tryptamine derivatives [53, 80, 90]. Potency in inducing hypolocomotion was found to be correlated with hypothermic potency, as HLA is known to be also mediated through 5-HT1AR activation [91].

In summary, the present study examined the pharmacology and behavioral effects of 5-methoxy-substituted tryptamines with a range of modifications on the amino position. 5-MeO-tryptamines analogs possess nanomolar affinity for 5-HT1AR and 5-HT2AR, which oppositely modulate the hallucinogenic response. Moreover, all tryptamines tested induced a remarkable hypothermic response in mice, an effect mediated by 5-HT1AR. Although some 5-MeO-tryptamines exhibited low HTR activity, all the tested compounds showed full agonism at 5-HT2AR. 5-MeO-tryptamine derivatives with bulkier substituents on the N position showed higher affinities for SERT and some tryptamines, especially 5-MeO-pyr-T, act as partial 5-HT releasers. Finally, and as mentioned before, the understanding of the pharmacological profile of this class of tryptamines will provide useful information for future studies in the field of drug therapy.

## Supplementary information

SUPPLEMENTARY INFORMATION

## Data Availability

The data that support the findings of this study are available from the corresponding author upon request.
