# Supplementary material for: Structure-activity relationships of serotonergic 5-MeO-DMT derivatives: insights into psychoactive and thermoregulatory properties
Source: Mol Psychiatry. 2024 Mar 14;29(8):2346–58. doi: 10.1038/s41380-024-02506-8 (PMC11412900; doi:10.1038/s41380-024-02506-8)
Supplement: Supplementary file 1 — SUPPLEMENTARY INFORMATION [file 41380_2024_2506_MOESM1_ESM.doc]

# SUPPLEMENTARY INFORMATION

**Structure-Activity Relationships of Serotonergic 5-MeO-DMT Derivatives: Insights into Psychoactive and Thermoregulatory Properties**

**Pol Puigseslloses^1,2^, Núria Nadal-Gratacós^1,2^, Gabriel Ketsela^2^, Nicola Weiss^1^, Xavier Berzosa^2^, Roger Estrada-Tejedor^2^, Mohammad Nazmul Islam^3^, Marion Holy^3^, Marco Niello^3,4^, David Pubill^1^, Jordi Camarasa^1^, Elena Escubedo^1^, Harald H. Sitte^3,5,6^, Raúl López-Arnau^1,*^**

^1^Department of Pharmacology, Toxicology and Therapeutic Chemistry, Pharmacology Section and Institute of Biomedicine (IBUB), Faculty of Pharmacy, University of Barcelona, 08028 Barcelona, Spain.

^2^Pharmaceutical Chemistry Group (GQF), IQS School of Engineering, Universitat Ramon Llull, 08017 Barcelona, Spain.

^3^ Institute of Pharmacology, Center for Physiology and Pharmacology, Medical University of Vienna, Wäehringerstrasse 13A, 1090 Vienna, Austria.

^4^ Genetics of Cognition Lab, Istituto Italiano di Tecnologia, Via Morego 30, 16163, Genova, Italy.

^5^Hourani Center for Applied Scientific Research, Al-Ahliyya Amman University, Amman, Jordan.

^6^ Center for Addiction Research and Science, Medical University Vienna, Waehringer Strasse 13A, 1090 Vienna, Austria.

^*^Corresponding author. Email: raullopezarnau@ub.edu

**SYNTHESIS OF 5-METHOXY-SUBSTITUTED TRYPTAMINES**

The synthesis and characterization of the novel 5-methoxy-substituted tryptamines (5-MeO-tryptamines) was performed through three steps, as previously described [1–3], with minor modifications, as seen in **Supplementary Figure 1**.

**Supplementary Figure 1.** Synthetic route for the 5-MeO-tryptamines.

**Step 1:** For the synthesis of the 5-methoxyindole-3-glyoxylamide, oxalyl chloride (5.18g, 1.20 eq.) was dissolved in dry diethyl ether (75 mL) and cooled to 0°C. 5-Methoxyindole (5.00 g, 1.00 eq.) was added portionwise. The bright orange precipitate thus formed was filtered after 30 min, washed with diethyl ether, dried in vacuo (3.59 g, 90% yield), and used without further purification.

**Step 2:** The compound obtained in Step 1 (1.50 g, 6.31 mmol, 1.0 eq) was added portionwise to a solution containing 15 mL of ice-cold THF and the corresponding amine (4.0 eq). The solution was heated under stirring in a sealed microwave flask for 15 min at 100ºC. The reaction mixture was cooled to room temperature and washed with 20mL of HCl 1M and brine to remove the excess of amine. The organic phase was separated, dried with MgSO_4_, and evaporated to yield a pale-yellow solid (70-90 % yield).

**Step 3:** In a three-necked round bottom flask with a cooler, LiAlH_4_ (0.87 g, 23.0 mmol, 7.0 eq) was suspended in dry THF (50 mL) under nitrogen atmosphere. The corresponding compound obtained in Step 2 (1.0 eq) was dissolved in dry THF (50 mL) and added dropwise at room temperature. The suspension was heated to reflux for 3 h. After cooling to room temperature, the excess of LiAlH_4_ was destroyed by careful addition of water and NaOH 1M. The salts were filtered over celite and washed with THF. Brine was added to the mixture and the organic layer was separated. The organic phase was dried with MgSO_4_, filtered and evaporated to yield a yellow oil (yield 60-80%).

**Salt formation:** The oily freebase (1g) was dissolved in 5 mL of acetone at room temperature. Afterwards, a concentrated solution of HCl/CPME diluted in acetone to 5% v/v, was added dropwise to the solution under stirring, until precipitation of a white solid (pH=5). The crystals were filtered with filter paper to yield the hydrochloride salt (40% yield). The identification of the synthesized 5-MeO-tryptamines was assessed by ^1^H-NMR, ^13^C-NMR, IR and MS.

Nuclear magnetic resonance spectra were recorded on a Varian 400-MR from Medical Systems (Palo Alto, USA) working at 400 MHz for ^1^H and 100.6 MHz for ^13^C and processed with MestreNova from MestraLab Research. 𝛿-values are stated in parts per million (ppm). The chemical shift is referenced to the residual proton signal of the deuterated solvent DMSO-*d6*: ^1^H NMR 𝛿 = 2.50 ppm and ^13^C NMR 𝛿 = 39.5 ppm. Infrared Spectra (IR) were performed and recorded in a Thermo Scientific Nicolet iS10 FTIR spectrometer with Smart iTR. Values are reported in cm^-1^ (wave numbers). Mass spectra (MS) were performed using an Agilent Technologies 5975 spectrometer (Electron Ionization).

***5-MeO-DMT:***

**^1^H NMR (400 MHz, DMSO-*d_6_*)** δ 10.83 (s, 1H), 10.55 (s, 1H), 7.25 (d, J = 8.8 Hz, 1H), 7.18 (d, J = 2.4 Hz, 1H), 7.15 (d, J = 2.4 Hz, 1H), 6.74 (dd, J = 8.7, 2.4 Hz, 1H), 3.78 (s, 3H), 3.27 (m, 2H), 3.09 (m, 2H), 2.82 (d, J = 4.9 Hz, 6H).

**13C NMR (100 MHz, DMSO-*d_6_*)** δ 153.17, 131.38, 127.07, 123.82, 112.19, 111.32, 108.73, 100.40, 56.56, 55.52, 41.97, 20.19 ppm.

**IR (KBr), v max:** 3296, 2954, 2664, 1630, 1492, 1463, 1213, 1166, 1076, 1037, 969, 800 cm-1

**m/z:** 218 (M+).

***5-MeO-DET:***

**^1^H NMR (400 MHz, DMSO-*d_6_*)** δ 10.83 (s, 1H), 10.33 (s, 1H), 7.25 (d, J = 9.2 Hz, 1H), 7.22 (d, J = 2.3 Hz, 1H), 7.14 (d, J = 2.4 Hz, 1H), 6.74 (dd, J = 8.7, 2.3 Hz, 1H), 3.78 (s, 3H), 3.21 (m, 2H), 3.18 (m, 4H), 3.11 (m, 2H), 1.26 (t, J = 7.3 Hz, 6H).

**^13^C NMR (100 MHz, DMSO-*d_6_*)** δ 153.59, 131.77, 127.49, 124.32, 112.62, 111.73, 109.35, 100.66, 55.90, 51.38, 46.30, 19.97, 8.89 ppm.

**IR (KBr), v max:** 3185, 2944, 2596, 1622, 1491, 1461, 1225, 1066, 1028, 925, 809 cm-1

**m/z:** 246 (M+).

***5-MeO-DiPT:***

**^1^H NMR (400 MHz, DMSO-*d_6_*)** δ 10.85 (s, 1H), 9.34 (s, 1H), 7.29 (d, J = 1.8 Hz, 1H), 7.26 (d, J = 8.7 Hz, 1H), 7.02 (d, J = 2.1 Hz, 1H), 6.75 (dd, J = 9.1, 2.0 Hz, 1H), 3.77 (s, 3H), 3.71 (m, 2H), 3.23 (m, 2H), 3.13 (m, 2H), 1.36 (d, J = 6.5 Hz, 6H), 1.34 (d, J = 6.5 Hz, 6H).

**^13^C NMR (100 MHz, DMSO-*d_6_*)** δ 153.15, 131.30, 126.95, 124.13, 112.25, 111.20, 109.15, 100.01, 55.34, 53.88, 47.02, 22.80, 18.11, 16.80 ppm.

**IR (KBr), v max:** 3163, 2938, 2632, 1627, 1491, 1433, 1221, 1069, 1031, 926, 803 cm-1 .

**m/z:** 274 (M+).

***5-MeO-MET:***

**^1^H NMR (400 MHz, DMSO-*d_6_*)** δ 10.89 (s, 1H), 10.86 (s, 1H), 7.25 (d, *J* = 8.8 Hz, 1H), 7.19 (d, *J* = 2.5 Hz, 1H), 7.18 (d, *J* = 2.5 Hz, 1H), 6.74 (dd, *J* = 8.8, 2.4 Hz, 1H), 3.78 (s, 3H), 3.35 – 3.03 (m, 6H), 2.78 (d, *J* = 5.0 Hz, 3H), 1.27 (t, *J* = 7.3 Hz, 3H).

**^13^C NMR (100 MHz, DMSO-*d_6_*)** δ 153.13, 131.33, 127.07, 123.75, 112.15, 111.29, 108.94, 100.35, 55.50, 54.35, 49.75, 38.14, 19.79, 8.80.

**IR (KBr), V max:** 3198, 2959, 2590, 1627, 1491, 1458, 1217, 1066, 1028, 926, 813 cm-1.

**m/z:** 232 (M+).

***5-MeO-MiPT:***

**^1^H NMR (400 MHz, DMSO-*d_6_*)** δ 10.89 (s, 1H), 10.86 (s, 1H), 7.25 (d, *J* = 8.8 Hz, 1H), 7.20 (t, *J* = 2.7 Hz, 2H), 6.73 (dd, *J* = 8.7, 2.4 Hz, 1H), 3.78 (s, 3H), 3.60 (hept, *J* = 6.6 Hz, 1H), 3.32 – 3.06 (m, 4H), 2.69 (s, 3H), 1.27 (dd, *J* = 28.9, 6.6 Hz, 6H).

**^13^C NMR (100 MHz, DMSO-*d_6_*)** δ 153.13, 131.31, 127.11, 123.78, 112.12, 111.27, 109.10, 100.39, 55.52, 55.50, 52.18, 34.12, 20.14, 16.81, 14.95.

**IR (KBr), V max:** 3197, 2934, 2583, 1621, 1491, 1460, 1218, 1079, 926, 814 cm-1 .

**m/z:** 246 (M+).

***5-MeO-EiPT:***

**^1^H NMR (400 MHz, DMSO-*d_6_*)** δ 10.87 (s, 1H), 10.68 (s, 1H), 7.25 (d, *J* = 8.8 Hz, 1H), 7.21 (dd, *J* = 18.2, 2.4 Hz, 2H), 6.73 (dd, *J* = 8.8, 2.4 Hz, 1H), 3.77 (s, 3H), 3.74 – 3.64 (m, 1H), 3.28 – 3.10 (m, *J* = 5.7, 4.9 Hz, 6H), 1.32 (dt, *J* = 13.8, 7.1 Hz, 9H).

**^13^C NMR (100 MHz, DMSO-*d_6_***) δ 153.15, 131.32, 127.07, 123.86, 112.18, 111.28, 109.24, 100.22, 55.46, 53.03, 48.80, 44.01, 20.45, 16.10, 16.00, 9.77.

**IR (KBr), V max:** 3185, 2934, 2574, 1623, 1491, 1438, 1214, 1075, 1026, 926, 813 cm-1.

**m/z:** 260 (M+).

***5-MeO-NiPT:***

**^1^H NMR (400 MHz, DMSO-*d_6_*)** δ 10.81 (s, 1H), 8.94 (s, 2H), 7.25 (d, *J* = 8.7 Hz, 1H), 7.21 (d, *J* = 2.5 Hz, 1H), 7.11 (d, *J* = 2.4 Hz, 1H), 6.74 (dd, *J* = 8.7, 2.4 Hz, 1H), 3.77 (d, *J* = 0.6 Hz, 3H), 3.33 – 3.26 (m, 1H), 3.07 (m, *J* = 13.1, 7.7 Hz, 4H), 1.26 (d, *J* = 6.5 Hz, 6H).

**^13^C NMR (100 MHz, DMSO-*d_6_*)** δ 153.12, 131.35, 127.14, 123.82, 112.14, 111.21, 109.29, 100.23, 55.48, 49.25, 44.25, 21.95, 18.59.

**IR (KBr), V max:** 3253, 2973, 2746, 1585, 1491, 1457, 1224, 1069, 926, 819.

**m/z:** 232 (M+).

***5-MeO-DALT:***

**^1^H NMR (400 MHz, DMSO-*d_6_*)** δ 11.21 (s, 1H), 10.82 (d, *J* = 2.6 Hz, 1H), 7.25 (d, *J* = 8.7 Hz, 1H), 7.18 (d, *J* = 2.5 Hz, 1H), 7.09 (d, *J* = 2.4 Hz, 1H), 6.74 (dd, *J* = 8.7, 2.4 Hz, 1H), 6.08 (ddt, *J* = 17.2, 10.2, 7.0 Hz, 2H), 5.66 – 5.50 (m, 4H), 3.83 (dd, *J* = 7.1, 4.7 Hz, 4H), 3.77 (s, 3H), 3.23 – 3.10 (m, 4H).

**^13^C NMR (100 MHz, DMSO-*d_6_*)** δ 153.16, 131.34, 127.38, 126.97, 124.99, 123.92, 112.25, 111.37, 108.73, 100.08, 55.42, 53.87, 51.31, 19.42.

**IR(KBr), V max:** 3166, 2940, 2505, 1593, 1492, 1461, 1223, 1076, 934, 804.

**m/z:** 270 (M+).

***5-MeO-MALT:***

**^1^H NMR (400 MHz, DMSO-*d_6_*)** δ 10.81 (s, 1H), 10.44 (s, 1H), 7.25 (d, *J* = 8.8 Hz 1H), 7.19 (d, *J* = 2.5 Hz, 1H), 7.11 (d, *J* = 2.4 Hz, 1H), 6.74 (dd, *J* = 8.8, 2.4 Hz, 1H), 6.01 (ddt, *J* = 17.2, 10.2, 7.1 Hz, 1H), 5.62 – 5.49 (m, 2H), 3.86 (m, 2H), 3.78 (s, 3H), 3.22 (m, 2H), 3.15 – 3.07 (m, 2H), 2.79 (s, 3H).

**^13^C NMR (100 MHz, DMSO-*d_6_*)** δ 153.14, 131.35, 127.57, 127.01, 124.89, 123.87, 112.19, 111.30, 108.68, 100.27, 56.97, 55.47, 54.38, 38.75, 19.81.

**IR(KBr), V max:** 3191, 2939, 2452, 1588, 1491, 1441, 1215, 1104, 938, 826.

**m/z:** 244 (M+).

***5-MeO-pyr-T:***

**^1^H NMR (400 MHz, DMSO-*d_6_*)** δ 10.80 (s, 1H), 10.57 (s, 1H), 7.25 (d, *J* = 8.8 Hz, 1H), 7.19 (d, *J* = 2.5 Hz, 1H), 7.12 (d, *J* = 2.5 Hz, 1H), 6.74 (dd, *J* = 8.8, 2.4 Hz, 1H), 3.78 (m, 3H), 3.56 (m, 2H), 3.36 (s, 2H), 3.13 – 2.96 (m, 4H), 2.05 – 1.83 (m, 4H).

**^13^C NMR (100 MHz, DMSO-*d_6_*)** δ 153.14, 131.36, 127.04, 123.81, 112.15, 111.27, 109.02, 100.36, 55.49, 54.02, 52.74, 22.69, 21.42.

**IR(KBr), V max:** 3189, 2940, 2591, 1623, 1490, 1435, 1216, 1175, 1030, 823.

**m/z:** 244 (M+).

**SOLUTIONS AND BUFFERS COMPOSITION**

**Krebs-HEPES-Buffer (KHB):** 10 mM HEPES, 120 mM NaCl, 3 mM KCl, 2 mM CaCl_2_·2H_2_O, 2 mM MgCl_2_·6H_2_O supplemented with 20 mM D-glucose, pH adjusted to 7.3.

**Physiological external solution:** 140 mM NaCl, 2.5 mM CaCl_2_, 3mM KCl, 2 mM MgCl_2_, 20 mM glucose, and 10 mM HEPES, pH adjusted to 7.3.

**Pipette solution:** 133 mM potassium gluconate, 5.9 mM NaCl, 1 mM CaCl2, 0.7 mM MgCl_2_, 10 mM HEPES, and 10 mM EGTA, pH adjusted to 7.2 with KOH.

**Binding buffers:**

- **for 5-HT2AR:** 50mM Tris, 4mM CaCl_2_, 0.1% ascorbic acid, pH=7.4 .
- **for 5-HT1AR:** 50 mM Tris, 10 mM MgSO_4_, 0.5 mM EDTA, 0.1% ascorbic acid, pH=7.4.
- **for hSERT:** 20 mM Tris, 120 mM NaCl, 3 mM KCl, 2 mM MgCl_2_, 1 mM EDTA, pH=7.4.

**Wash buffers:**

- **for 5-HT1AR and 5-HT2AR**: 50 mMTris, pH=7.4.
- **for hSERT:** 120 mM NaCl, 2 mM MgCl_2_, 10 mM Tris, pH=7.4.

**CELL CULTURE AND MEMBRANE PREPARATIONS**

The generation and maintenance of HEK293 cells stably expressing the yellow-fluorescent protein (YFP)-tagged version of the human isoform of SERT (hSERT) (Perkin Elmer, Waltham, MA, USA) was carried out as described previously [4]. For uptake inhibition and batch release assays, cells were seeded onto previously poly-D-lysine (PDL)-coated 96-well plates at a density of 0.036 million cells per well [5]. For superfusion release assays, cells were seeded onto a previously coated with PDL 6 channel µ-slide at a density of 0.08 million cells per channel. Prior to patching, cells were grown in DMEM containing 10% FBS, Zeocin (150 µg/mL) and Blasticidin (6 µg/mL) for positive selection of transporter-expressing clones. The cells were seeded at a low density on PDL-coated 35 mm plates 24-48 hr prior to patching. For membrane preparation, HEK293 cells expressing hSERT transporters were harvested from 80–90% confluent dishes as described elsewhere [6]. Protein concentration was determined using the Bio-RadProtein Reagent (Bio Rad Laboratories, Hercules, CA, USA). Membrane preparations were stored at −80°C until use. Generation and maintenance of CHO-K1 cells expressing the human isoform of 5-HT2AR (h5-HT2AR) was carried out as described in the manufacturer’s protocol (Perkin Elmer, Waltham, MA, USA). Routine DAPI-Staining assays tested negative in mycoplasma contamination.

**BIBLIOGRAPHY**

1. Vermeulen ES, van Smeden M, Schmidt AW, Sprouse JS, Wikström H v., Grol CJ. Novel 5-HT7 receptor inverse agonists. Synthesis and molecular modeling of arylpiperazine- and 1,2,3,4-tetrahydroisoquinoline-based arylsulfonamides. J Med Chem. 2004;47:5451–5466.

2. Brandt SD, Tirunarayanapuram SS, Freeman S, Dempster N, Barker SA, Daley PF, et al. Microwave-accelerated synthesis of psychoactive deuterated N,N-dialkylated-[α,α,β,β-d4]-tryptamines. J Labelled Comp Radiopharm. 2008;51:423–429.

3. Speeter ME, Anthony WC. The action of oxalyl chloride on indoles: a new approach to tryptamines. J Am Chem Soc. 1954;76:6208–6210.

4. Mayer FP, Wimmer L, Dillon-Carter O, Partilla JS, Burchardt N v., Mihovilovic MD, et al. Phase I metabolites of mephedrone display biological activity as substrates at monoamine transporters. Br J Pharmacol. 2016:2657–2668.

5. Nadal-Gratacós N, Alberto-Silva AS, Rodríguez-Soler M, Urquizu E, Espinosa-Velasco M, Jäntsch K, et al. Structure–Activity Relationship of Novel Second-Generation Synthetic Cathinones: Mechanism of Action, Locomotion, Reward, and Immediate-Early Genes. Front Pharmacol. 2021;12.

6. Nadal-Gratacós N, Lleixà E, Gibert-Serramià M, Estrada-Tejedor R, Berzosa X, Batllori X, et al. Neuropsychopharmacology of Emerging Drugs of Abuse: meta-and para-Halogen-Ring-Substituted α-PVP (“flakka”) Derivatives. Int J Mol Sci. 2022;23.

**SUPPLEMENTARY FIGURES**

**Supplementary Figure 2.** Competition binding curves. **a** [^3^H]8-OH-DPAT binding to 5-HT_1A_ receptor. **b** [^3^H]ketanserin binding to 5-HT_2A_ receptor. **c** [^3^H]imipramine binding to hSERT. Data are expressed as percentage of control binding (absence of tryptamine) and are the mean from at least three independent experiments.

**Supplementary Figure 3**. Correlation (P<0.01, R^2^=0.6112) between molecular volume (Å^3^) and the experimental Ki values (µM) of 5-MeO-tryptamines for hSERT.

**Supplementary Figure 4. a-f** Effects of 5-MeO-MALT, 5-MeO-DALT, 5-MeO-NIPT, 5-MeO-MIPT, 5-MeO-EIPT and 5-MeO-DIPT on transport-mediated batch release of preloaded [^3^H]5-HT from HEK293 cells stably expressing SERT. **g-h** Paroxetine and pCA were used as control substances. * p<0.05, ** p<0.01, *** p<0.001 vs release in absence of monensin (mixed-effects model employing Šidák’s correction). Data are expressed as means ± SD for N=5 independent experiments.

**Supplementary Figure 5.** Dose-response curves for HTR of all 5-MeO-tryptamines studied. Data are expressed as means ± SD. N=8-10 mice per group.

**Supplementary Figure 6.** Correlation (P<0.05, R^2^=0.4194) between EC_50_ of 5-HT2AR functional assay (calcium mobilization) and E_max_ of HTR.

**Supplementary Figure 7.** Correlation (P<0.05, R^2^=0.4082) between molecular volume (Å^3^) and E_max_ HTR.

**Supplementary Figure 8.** Number of head twitches after tryptamine injection (i.p., 10 mg/kg) with or without 5-HT1AR or 5-HT2AR antagonist pretreatment, WAY100635 (s.c., 1 mg/kg; WAY) or ketanserin (s.c., 1 mg/kg; KS), respectively. Data are presented as means ± SD. * p<0.05, ** p<0.01, *** p<0.001 vs control group. # p<0.05, ## p<0.01, ### p<0.001 vs group receiving only tryptamine (Tuckey’s test). N=8-10 mice per group. For clarity purposes, 5-MeO-tryptamine bars effect were duplicated in each graph. However, all experimental groups showed in each graph were tested at the same time in order to reduce the number of animals used.

**Supplementary Figure 9.** Dose-response curves for core body temperature measurements. Data are expressed as means ± SD. N=8-10 mice per group.

**Supplementary Figure 10.** Core body temperature measured after 60 minutes of tryptamine injection (i.p. 10 mg/kg) with or without 5-HT1AR antagonist pretreatment, WAY100635 (s.c., 1 mg/kg) (WAY). Data are shown as means ± SD. * p<0.05, ** p<0.01, *** p<0.001 vs control group. # p<0.05, ## p<0.01, ### p<0.001 vs group receiving only tryptamine (ANOVA with Tuckey’s test or Kruskal-Wallis with Dunn’s test). N=8-10 mice per group.

**Supplementary Figure 11.** Dose-response curves for HLA of all 5-MeO-tryptamines studied. Data are expressed as means ± SD. N=8-10 mice per group.

**Supplementary Figure 12.** Correlation (P<0.01, R^2^=0.5904) between potencies at inducing hypothermia and hypolocomotion (ED_50_).

**STATISTICAL DATA**

**Supplementary Table 1. Statistical results for transport-mediated batch release assays (mixed-effects model) and transport-mediated superfusion release assays (one-way ANOVA, post-hoc Tukey-Kramer’s test).**

| **Batch release assays** | | | | | | | |
| --- | --- | --- | --- | --- | --- | --- | --- |
| *Compound* | *F time (DFn, DFd)* | *P value* | *F treatment (DFn, DFd)* | | *P value* | *F interaction (DFn, DFd)* | *P value* |
| **5-MeO-DMT / 5-MeO-DMT + Mon** | F_(1.479,27.36)_ = 229.9 | < 0.001 | F_(3,20)_ = 43.89 | | <0.001 | F_(12,74)_ = 33.73 | <0.001 |
| **5-MeO-MET / 5-MeO-MET + Mon** | F_(1.595,25.52)_ = 158.0 | <0.001 | F_(3,16)_ = 24.68 | | <0.001 | F_(12,64)_ = 29.45 | <0.001 |
| **5-MeO-DET / 5-MeO-DET + Mon** | F_(1.389,23.96)_ = 193.9 | <0.001 | F_(3,18)_ = 38.63 | | <0.001 | F_(12,69)_ = 31.96 | <0.001 |
| **5-MeO-pyr-T / 5-MeO-pyr-T + Mon** | F_(1.339,25.10)_ = 63.41 | <0.001 | F_(3,19)_ = 37.22 | | <0.001 | F_(12,75)_ = 25.88 | <0.001 |
| **5-MeO-MALT / 5-MeO-MALT + Mon** | F_(2.606,41.70)_ = 97.55 | <0.001 | F_(3,16)_ = 7.245 | | <0.01 | F_(12,64)_ = 9.184 | <0.001 |
| **5-MeO-DALT / 5-MeO-DALT + Mon** | F_(2.098,33.58)_ = 111.5 | <0.001 | F_(3,16)_ = 10.26 | | <0.001 | F_(12,64)_ = 13.43 | <0.001 |
| **5-MeO-NIPT / 5-MeO-NIPT + Mon** | F_(2.680,42.89)_ = 38.27 | <0.001 | F_(3,16)_ = 1.698 | | >0.05 | F_(12,64)_ = 4.852 | <0.001 |
| **5-MeO-MIPT / 5-MeO-MIPT + Mon** | F_(2.220,35.51)_ = 88.28 | <0.001 | F_(3,16)_ = 19.80 | | <0.001 | F_(12,64)_ = 16.38 | <0.001 |
| **5-MeO-EIPT / 5-MeO-EIPT + Mon** | F_(1.908,30.52)_ = 304.3 | <0.001 | F_(3,16)_ = 58.63 | | <0.001 | F_(12,64)_ = 40.65 | <0.001 |
| **5-MeO-DIPT / 5-MeO-DIPT + Mon** | F_(1.747,27.96)_ = 12.57 | <0.001 | F_(3,16)_ = 1.781 | | >0.05 | F_(12,64)_ = 1.191 | >0.05 |
| **Paroxetine** ª **/ Paroxetine** ª **+ Mon** | F_(1.148, 13.78)_ = 20.03 | <0.001 | F_(1,12)_ = 3.189 | | >0.05 | F_(4,48)_ = 2.257 | >0.05 |
| **PCA** ª **/ PCA** ª **+ Mon** | F_(1.877, 22.52)_ = 1203 | <0.001 | F_(1,12)_ = 332.9 | | <0.001 | F_(4,48)_ = 308.5 | <0.001 |
| **Superfusion release assays with 5-MeO-pyr-T** | | | | | | | |
| *F treatment (DFn, DFd)* | | | | *P value* | | | |
| F_(7,31)_ = 407.2 | | | | <0.001 | | | |

ª Control compounds.

**Supplementary Table 2.** Statistical data (One-way ANOVA with Dunnet’s test or Kruskall-Wallis with Dunn’s test) for HTR experiments and core body temperature measurements

|  | **HTR** | | **Core body temperature** | |
| --- | --- | --- | --- | --- |
| *Compound* | *H treatment (DF)* | *P value* | *F treatment (DFn, DFd) or H treatment (DF)* | *P value* |
| 5-MeO-DMT | H _(5)_ = 51.21 | <0.001 | H _(5)_ = 29.61 | <0.001 |
| 5-MeO-MET | H _(5)_ = 46.86 | <0.001 | F _(5, 54)_ = 8.900 | <0.001 |
| 5-MeO-DET | H _(5)_ = 34.39 | <0.001 | F _(5, 52)_ = 50.77 | <0.001 |
| 5-MeO-pyr-T | H _(5)_ = 27.76 | <0.001 | F _(5, 49)_ = 30.38 | <0.001 |
| 5-MeO-MALT | H _(5)_ = 40.83 | <0.001 | H _(5)_ = 33.26 | <0.001 |
| 5-MeO-DALT | H _(5)_ = 35.87 | <0.001 | F _(5, 52)_ = 44.73 | <0.001 |
| 5-MeO-NIPT | H _(5)_ = 14.66 | 0.01 | F _(5, 54)_ = 61.11 | <0.001 |
| 5-MeO-MIPT | H _(5)_ = 36.04 | <0.001 | F _(5, 54)_ = 29.09 | <0.001 |
| 5-MeO-EIPT | H _(5)_ = 29.43 | <0.001 | H _(5)_ = 36.08 | <0.001 |
| 5-MeO-DIPT | H _(5)_ = 31.92 | <0.001 | F _(5, 54)_ = 24.11 | <0.001 |

**Supplementary Table 3.** Statistical data (one-way ANOVA with Tuckey’s test or Kruskal-Wallis with Dunn’s test) for HTR experiments using 5-HT antagonists (ketanserine and WAY100635) and core body temperature measurements using 5-HT antagonists.

|  | **HTR (ketanserin)** | | **HTR (WAY100635)** | | **Core body temperature (WAY 100635)** | |
| --- | --- | --- | --- | --- | --- | --- |
| *Compound* | *F treatment (DFn, DFd)* | *P value* | *F treatment (DFn, DFd)* | *P value* | *F treatment (DFn, DFd) or H treatment (DF)* | *P value* |
| 5-MeO-DMT | F _(3, 36)_ = 27.40 | <0.001 | F _(3, 35)_ = 45.37 | <0.001 | H _(3)_ = 18.05 | <0.001 |
| 5-MeO-MET | F _(3, 36)_ = 74.75 | <0.001 | F _(3, 36)_ = 53.31 | <0.001 | F _(3, 36)_ = 9.724 | <0.001 |
| 5-MeO-DET | F _(3, 35)_ = 26.75 | <0.001 | F _(3, 34)_ = 58.84 | <0.001 | F _(3, 35)_ = 40.58 | <0.001 |
| 5-MeO-pyr-T | F _(3, 35)_ = 13.56 | <0.001 | F _(3, 36)_ = 40.04 | <0.001 | F _(3, 34)_ = 75.42 | <0.001 |
| 5-MeO-MALT | F _(3, 36)_ = 130.4 | <0.001 | F _(3, 33)_ = 376.7 | <0.001 | F _(3, 36)_ = 6.956 | <0.01 |
| 5-MeO-DALT | F _(3, 36)_ = 38.17 | <0.001 | F _(3, 35)_ = 54.69 | <0.001 | F _(3, 34)_ = 4.106 | 0.01 |
| 5-MeO-NIPT | F _(3, 36)_ = 23.72 | <0.001 | F _(3, 36)_ = 221.2 | <0.001 | H _(3)_ = 23.42 | <0.001 |
| 5-MeO-MIPT | F _(3, 36)_ = 72.86 | <0.001 | F _(3, 36)_ = 43.71 | <0.001 | H _(3)_ = 21.57 | <0.001 |
| 5-MeO-EIPT | F _(3, 36)_ = 27.32 | <0.001 | F _(3, 36)_ = 54.77 | <0.001 | F _(3, 36)_ = 7.265 | <0.001 |
| 5-MeO-DIPT | F _(3, 36)_ = 75.45 | <0.001 | F _(3, 36)_ = 60.64 | <0.001 | H _(3)_ = 17.34 | <0.001 |

**Supplementary Table 4.** Statistical data (one-way ANOVA with Dunnet’s test or Kruskal-Wallis with Dunn’s test) for HLA.

|  | **HLA** | |
| --- | --- | --- |
| *Compound* | *F treatment (DFn, DFd) or H treatment (DF)* | *P value* |
| 5-MeO-DMT | H _(5)_ = 21.66 | <0.001 |
| 5-MeO-MET | F _(5, 53)_ = 23.31 | <0.001 |
| 5-MeO-DET | F _(5, 50)_ = 3.864 | <0.01 |
| 5-MeO-pyr-T | F _(5, 54)_ = 12.72 | <0.001 |
| 5-MeO-MALT | F _(5, 54)_ = 18.44 | <0.001 |
| 5-MeO-DALT | F _(5, 51)_ = 21.24 | <0.001 |
| 5-MeO-NIPT | F _(5, 53)_ = 32.07 | <0.001 |
| 5-MeO-MIPT | F _(5, 53)_ = 20.59 | <0.001 |
| 5-MeO-EIPT | F _(5, 54)_ = 22.29 | <0.001 |
| 5-MeO-DIPT | H _(5)_ = 44.35 | <0.001 |
